# A Novel 2-DOF PIDA control strategy with GCRA-based parameter optimization for electric furnace temperature control

**DOI:** 10.1371/journal.pone.0334594

**Published:** 2025-10-14

**Authors:** Erdal Eker, Davut Izci, Serdar Ekinci, Fahmi Elsayed, Mohammad Salman

**Affiliations:** 1 Vocational School of Social Sciences, Muş Alparslan University, Muş, Turkey; 2 Department of Electrical and Electronics Engineering, Bursa Uludag University, Bursa, Turkey; 3 Applied Science Research Center, Applied Science Private University, Amman, Jordan; 4 Department of Computer Engineering, Bitlis Eren University, Bitlis, Turkey; 5 College of Engineering and Technology, American University of the Middle East, Egaila, Kuwait; SR University, INDIA

## Abstract

Accurate and energy-efficient temperature regulation in electric furnace systems remains a challenging control problem due to nonlinear dynamics, significant thermal inertia, and inevitable time delays. Conventional proportional–integral–derivative (PID) and PID–acceleration (PIDA) controllers, though widely used, often exhibit degraded performance under such conditions, particularly when implemented in a single-degree-of-freedom. To address these limitations, this study proposes, for the first time, a two-degree-of-freedom (2-DOF) PIDA controller tailored for electric furnace temperature control. The controller structure allows independent tuning of set-point tracking and disturbance rejection by introducing separate feedforward paths in the proportional and derivative channels while maintaining integral and acceleration actions on the error signal. To optimize the controller parameters, the recently developed greater cane rat algorithm (GCRA) is employed for the first time in this context. A novel adaptive objective function (combining normalized overshoot, normalized settling time, and cumulative tracking error) guides the tuning process to achieve a balanced improvement in both transient and steady-state performance. The proposed GCRA-based 2-DOF PIDA controller is evaluated through extensive simulations and compared against state-of-the-art metaheuristic tuning approaches, including polar fox optimization (PFA), hiking optimization (HOA), success-history based adaptive differential evolution with linear population size reduction (L-SHADE), and particle swarm optimization (PSO), as well as several benchmark furnace control methods. Results demonstrate that the proposed method consistently achieves faster settling times, reduced overshoot, and near-zero steady-state error, while maintaining robustness under external disturbances and measurement noise. For instance, in the nominal case, the method yields an overshoot of 1.8382% and a settling time of 3.4542 s, outperforming PFA, HOA, L-SHADE, and PSO. Robustness tests under load disturbances and measurement noise confirm stable operation with minimal performance degradation, achieving less than 2.5% overshoot and under 4 s settling time across all evaluated scenarios. These findings highlight the potential of the GCRA-based 2-DOF PIDA controller as a high-precision and energy-efficient solution for temperature regulation in industrial time-delay systems.

## 1. Introduction

Temperature regulation in electric furnace systems is a critical task in many industrial applications, ranging from materials processing to precision manufacturing [1–4]. Achieving high‐performance control in these systems is challenging due to their nonlinear dynamics, significant thermal inertia, and unavoidable time delays [5]. Such delays, often caused by sensor lag and slow heat transfer, can degrade both stability and responsiveness if not properly addressed. Inaccurate or sluggish temperature control not only reduces product quality but can also lead to excessive energy consumption and operational inefficiencies.

Conventional proportional–integral–derivative (PID) controllers have been widely employed for furnace temperature regulation due to their simple structure and ease of implementation [6–9]. For instance, Grassi and Tsakalis [10] applied frequency loop-shaping techniques to PID tuning for diffusion furnaces, achieving improved stability and tracking accuracy. However, their performance tends to deteriorate in the presence of pure time delays and process nonlinearities, particularly when a single‐degree‐of‐freedom (1‐DOF) structure is used. In these cases, proportional, integral, and derivative actions are applied to the same error signal, making it difficult to independently tune the set‐point tracking and disturbance‐rejection behaviors [11]. Extensions such as proportional–integral–derivative–acceleration (PIDA) controllers [12–18] offer improved transient performance by adding an acceleration term, yet most reported designs still retain a 1‐DOF configuration, limiting flexibility in shaping the closed‐loop response.

To overcome these shortcomings, this study introduces a two‐degree‐of‐freedom (2‐DOF) PIDA controller specifically tailored for the temperature regulation of time‐delay systems. To the best of the authors’ knowledge, this is the first reported application of such a controller structure to an electric furnace temperature system. By incorporating separate feedforward weights in the proportional and derivative paths while maintaining integral and acceleration actions on the raw error, the proposed design enables independent tuning of reference tracking and disturbance rejection. This structural flexibility allows the controller to achieve rapid, low‐overshoot responses without sacrificing steady‐state accuracy or robustness to disturbances.

An equally important contribution of this work lies in the tuning strategy. Controller parameters are optimized using the greater cane rat algorithm (GCRA) [19], a recent nature‐inspired metaheuristic that models the adaptive foraging behavior of cane rats. Although GCRA has shown promise in generic optimization tasks, this is the first time it has been applied to tune a 2‐DOF PIDA controller for temperature regulation in time‐delay systems. The tuning process is driven by a novel adaptive objective function that combines normalized overshoot, normalized settling time, and cumulative tracking error [20] into a single metric. This formulation ensures balanced improvements in both transient and steady‐state performance, which is particularly important in thermal processes where overshoot and prolonged settling can be costly.

The effectiveness of the proposed GCRA‐based 2‐DOF PIDA controller is rigorously validated through comparative studies against several recent and established tuning methods, including metaheuristic optimizers such as polar fox optimization algorithm [21], hiking optimization algorithm [22] (selected as representative of recently developed strategies demonstrating strong exploration–exploitation balance), success-history based adaptive differential evolution with linear population size reduction [23] (recognized as a winner in CEC benchmark competitions), and particle swarm optimization [24] (the most widely applied swarm-based technique), as well as reported benchmark approaches like artificial rabbits optimization-based filtered PID [6], modified electric eel foraging optimizer-based filtered PID [25], genetic algorithm-based PID [26] and Ziegler-Nichols-based PID [27] controllers. The results demonstrate that the proposed method consistently achieves faster settling, reduced overshoot, and near‐zero steady‐state error, while maintaining robustness under external disturbances and measurement noise. In summary, the main contributions of this study are:

1. The first‐ever design and application of a 2‐DOF PIDA controller for temperature regulation in electric furnace systems with time delay.
2. The first use of the GCRA to tune such a controller structure, leveraging its balance between exploration and exploitation for precise parameter optimization.
3. A novel adaptive objective function that holistically balances transient and steady‐state performance metrics.
4. A comprehensive performance evaluation, including robustness analysis and comparisons with state‐of‐the‐art methods, demonstrating the superiority of the proposed approach.

The remainder of this paper is organized as follows. Section 2 provides a detailed overview of the GCRA, explaining its biological inspiration, mathematical formulation, and adaptive search behavior. Section 3 describes the electric furnace temperature system under consideration, emphasizing its nonlinear dynamics and inherent time‐delay characteristics. Section 4 presents the design of the proposed 2‐DOF PIDA controller, including its structural configuration and the novel GCRA‐based parameter tuning process. Section 5 reports the simulation setup, performance evaluation metrics, and comparative results against established and recent optimization‐based control approaches, along with robustness assessments. Finally, Section 6 summarizes the main findings, outlines potential industrial implications, and suggests future research directions.

## 2. Overview of greater cane rat algorithm (GCRA)

The greater cane rat algorithm (GCRA) [19] draws inspiration from the foraging patterns of greater cane rats, both in and out of their breeding season, to solve complex optimization problems. By emulating the way these animals search for food (alternating between wide-ranging exploration and focused exploitation) the GCRA effectively balances the need to discover new regions of the search space with the need to refine promising solutions.

The process begins by scattering an initial population of candidate solutions, denoted as X, across the search domain. Each individual rat’s position $x_{i,j}$ in the $j^{th}$ dimension is determined by $x_{i,j}=rand\times \left({UB}_{i}-{LB}_{i}\right)+{LB}_{i}$ where ${UB}_{i}$ and ${LB}_{i}$ define the permitted upper and lower bounds, and rand is a uniform random number between 0 and 1. This random initialization ensures diversity, laying the groundwork for a robust search.

Within the rat community, a dominant male (who has learned the best food locations) guides the others. During exploitation, each rat updates its position by averaging its current location with that of the leader:


$${x_{new}}_{i,j}=\text{0}.\text{7}\times \frac{x_{i,j}+x_{k,j}}{\text{2}}$$ (1)

where $x_{k,j}$ is the dominant male’s coordinate.

The algorithm toggles between exploration and exploitation depending on a control threshold (ρ=0.5). In the exploration phase, rats venture out to uncover new feeding grounds, adjusting their positions according to ${x_{new}}_{i,j}=x_{i,j}+C\times \left(x_{k,j}-r\times x_{i,j}\right)$ and then selecting between two update rules based on whether the newly evaluated objective value $F_{i}^{new}$ improves upon the current value $F_{i}$:


$$X_{i}=\left\{ \begin{array}{c} x_{i,j}+C\times \left(x_{k,j}-\alpha \times x_{k,j}\right),\;F_{i}^{new}<F_{i}\\ x_{i,j}+C\times \left(x_{m,j}-\beta \times x_{k,j}\right),\;otherwise \end{array}\right.\;$$ (2)

where $x_{m,j}$ is the position of a randomly chosen female rat, and the parameters C, r, α, and β modulate the step sizes in response to the abundance or scarcity of resources.

When the population enters mating season, males concentrate their search around potential mates rather than roaming widely. In this intensification phase, positions are refined as ${x_{new}}_{i,j}=x_{i,j}+C\times \left(x_{k,j}-\mu \times x_{m,j}\right)$ with μ randomly selected between 1 and 4 to mirror varying litter sizes and to focus the search even more narrowly.

By alternating among these behaviors (random dispersal, leader-guided convergence, and mate-focused intensification) the GCRA maintains a dynamic equilibrium between seeking unexplored regions and honing in on promising solutions. This structured mimicry of cane rat behavior endows the algorithm with both versatility and resilience, making it well suited to tackle a wide range of challenging optimization tasks.

**Algorithm 1. Pseudocode of GCRA**

**Input:** X – initial population (size popSize), max_iter (maximum number of iterations), LB, UB (lower and upper bounds for each dimension), ρ (exploration/exploitation threshold)

**Output:** Gbest – best solution found

 1. **Initialization**

   1.1 iter ← 0

   1.2 For i = 1 to popSize

    For j = 1 to D

     xᵢⱼ ← rand × (UBⱼ – LBⱼ) + LBⱼ

    End For

    Fᵢ ← EvaluateObjective(xᵢ)

   End For

 2. **Select initial leader**

   Gbest ← solution with lowest Fᵢ

   k ← index of the dominant male

 3. **Initial exploitation**

   For i = 1 to popSize, i ≠ k

    Xᵢ ← 0.7 × (Xᵢ + Xₖ) ÷ 2

    Fᵢ ← EvaluateObjective(Xᵢ)

   End For

   Update Gbest and k

 4. **Main loop**

   While iter <max_iter

 4.1 Compute C,r,α, β, μ

 4.2 For i = 1 to popSize

If rand(0,1) <ρ Then

 **Exploration**

 temp ← Xᵢ + C × (Xₖ – r × Xᵢ)

 F_temp ← EvaluateObjective(temp)

 If F_temp < Fᵢ Then

  X_new ← Xᵢ + C × (Xₖ – α × Xₖ)

 Else

  m ← random index ≠ i

  X_new ← Xᵢ + C × (X_ₘ_ – β × Xₖ)

 End If

Else

 **Exploitation**

 If inMatingSeason(iter) Then

  m ← random index ≠ i

  X_new ← Xᵢ + C × (Xₖ – μ × X_ₘ_)

 Else

  X_new ← 0.7 × (Xᵢ + Xₖ) ÷ 2

 End If

End If

 4.3 Boundary check and fitness update

 X_new ← EnforceBounds(X_new, LB, UB)

 F_new ← EvaluateObjective(X_new)

 4.4 Greedy acceptance

 If F_new < Fᵢ Then

  Xᵢ ← X_new

  Fᵢ ← F_new

 End If

 End For

 4.5 Update Gbest and k

 Gbest ← solution with lowest Fᵢ

 k ← index of dominant male

 4.6 iter ← iter + 1

End While

 5.**Return** Gbest

## 3. Mathematical modeling of electric furnace control system

**Fig 1:**
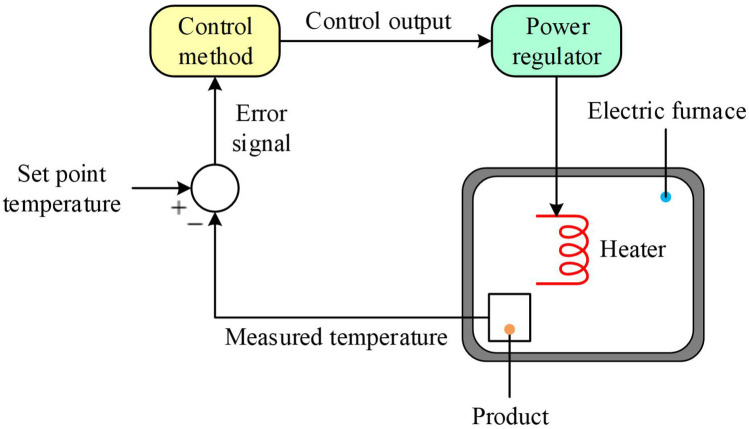
Block diagram of the electric furnace temperature control system.

Fig 1 illustrates the feedback architecture employed to regulate the temperature of the electric furnace. The system continuously measures the internal temperature and compares it to a desired set-point. Any discrepancy generates an error signal, which the controller processes to determine the appropriate adjustment. This control action is sent to the power regulator, which then modulates the heater’s input power to drive the temperature back toward the set-point. Such a closed-loop arrangement compensates for thermal inertia and external disturbances, maintaining the furnace at its target temperature.

We represent the furnace dynamics with a second-order transfer function augmented by an exact dead-time term (second-order plus dead time):


$$G_{plant}(s)=\frac{b}{a_{\text{2}}s^{\text{2}}+a_{\text{1}}s+a_{\text{0}}}e^{-Ds}$$ (3)

Here, the coefficients $a_{\text{2}}$, $a_{\text{1}}$, and $a_{\text{0}}$ capture the furnace’s thermal capacity and heat-transfer characteristics, b scales the steady-state gain, and D denotes the pure time delay arising from sensor lag and the furnace’s thermal response. To remain consistent with established studies [25–27], we adopt the numerical values of $a_{\text{0}}=\text{0}.\text{2}$, $a_{\text{1}}=\text{1}.\text{1}$, $a_{\text{2}}=\text{1}$, b=0.15 and D=1.5.

By retaining the exact exponential term $e^{-Ds}$ rather than approximating it with a Padé expansion [28], our model preserves the true non-rational nature of the delay. This fidelity is essential for accurately predicting phase lag and gain attenuation introduced by the dead time, which in turn leads to more reliable stability-margin assessments and robust controller designs. In subsequent sections, the controller synthesis will explicitly account for this pure delay, ensuring that both phase-margin specifications and disturbance-rejection requirements are met without the approximation errors that a Padé model would introduce.

## 4. Proposed control methodology: Novel objective function and GCRA based 2-DOF PIDA controller

**Fig 2:**
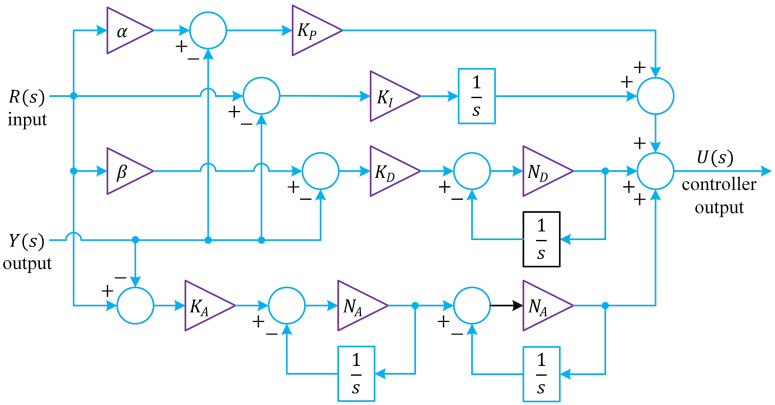
Block diagram of 2-DOF PIDA controller.


A proportional–integral–derivative–acceleration (PIDA) controller combines the classical PID actions with an additional acceleration term to improve both transient response and disturbance rejection [29]. Building on this principle, the two‐degree‐of‐freedom (2 DOF) PIDA controller is realized by four parallel signal paths, each of which corresponds directly to one of the additive terms in the transfer‐function expression (Eq. (4)) and is depicted in the block diagram of Fig 2. At the outset, the proportional branch applies a feedforward weight α to the reference input R(s) before comparing it with the measured output Y(s). As indicated by the first term of Eq. (4), this weighted error αR(s)−Y(s) is then scaled by the proportional gain $K_{P}$, thereby governing how aggressively the controller tracks set‐point changes.

$$U(s)=K_{P}\left[\alpha R(s)-Y(s)\right]+\frac{K_{I}}{s}\left[R(s)-Y(s)\right]+K_{D}\frac{N_{D}s}{s+N_{D}}\left[\beta R(s)-Y(s)\right]+K_{A}{\left(\frac{N_{A}s}{s+N_{A}}\right)}^{\text{2}}\left[R(s)-Y(s)\right]$$ (4)

In parallel, the integral branch addresses any residual steady‐state error by passing the unweighted discrepancy R(s)−Y(s) through the integrator $K_{I}$. This action, represented by the second term in Eq. (4), ensures that the long‐term average error is driven to zero. The third path implements a filtered derivative action with its own feedforward weight β. As shown in Eq. (4), the error βR(s)−Y(s) is first amplified by $K_{D}$ and then filtered by the first‐order term $\left(N_{D}s\right)/\left(s+N_{D}\right)$. In accordance with the fourth term of Eq. (4), the raw error R(s)−Y(s) is multiplied by the acceleration gain $K_{A}$ and subsequently passed twice through the same first‐order filter $\left(N_{A}s\right)/\left(s+N_{A}\right)$, thereby realizing a second‐order lead characteristic. This “acceleration” effect contributes additional phase margin and sharpens the controller’s transient response without unduly exciting measurement noise. All four branch outputs are then summed at the final junction on the right side of Eq. (4) to produce the overall control signal U(s). By incorporating independent set‐point weights α and β in the proportional and derivative loops while retaining integral and acceleration actions on the raw error, the structure depicted in Fig 2 faithfully implements the composite transfer function of Eq. (4) and enables separate tuning of reference tracking and disturbance‐rejection characteristics.

The proposed 2-DOF PIDA controller distinguishes itself from both conventional PID [30] and single‐degree‐of‐freedom PIDA [31] schemes by offering dedicated tuning channels for set‐point tracking and disturbance rejection, while remaining straightforward to implement [32]. In a standard PID, proportional and derivative actions indiscriminately react to any error, and even in a single‐DOF PIDA all four corrective branches (proportional, integral, derivative, and acceleration) operate on the same error signal, precluding independent shaping of reference and disturbance responses. By contrast, the 2‐DOF PIDA leverages separate feedforward weights in its proportional and derivative paths, so that the aggressiveness of set‐point changes can be adjusted without compromising the controller’s ability to suppress disturbances or eliminate steady‐state error. From a practical perspective, the parallel‐branch realization aligns naturally with digital control architectures or analog circuitry, and the inclusion of first‐order filters in the derivative and acceleration channels mitigates measurement noise. Consequently, the 2‐DOF PIDA delivers enhanced transient performance, reduced overshoot, and robust disturbance handling in a form that remains accessible for industrial deployment.

The control performance of the proposed methodology was quantified by the adaptive objective function (OF) defined in Eq. (5), which combines three key measures of time‐domain behavior into a single scalar metric. Specifically, the normalized percent overshoot (OS) was weighted by $\rho_{\text{1}}$, the normalized settling time (ST) by $\rho_{\text{2}}$, and the cumulative tracking error e(t)=r(t)−y(t) by the remaining weight $\left(\text{1}-\rho_{\text{1}}-\rho_{\text{2}}\right)$. In all simulations, the coefficients were set to $\rho_{\text{1}}=\text{0}.\text{1}\text{5}$ and $\rho_{\text{2}}=\text{0}.\text{0}\text{5}$, and a prediction horizon (simulation time) of $t_{f}=\text{5}\text{0}$ s was adopted. This adaptive objective function can be considered as a modified versin of integral of absolute error (IAE) [20] which is described by the integral term of Eq. (5). During each trial, a step change in the reference temperature from 200 °C to 210 °C was applied when computing the OF, ensuring that both transient peaks and steady‐state deviations were captured.


$$OF=\rho_{\text{1}}\cdot OS+\rho_{\text{2}}\cdot ST+\left(\text{1}-\rho_{\text{1}}-\rho_{\text{2}}\right)\int_{\text{0}}^{t_{f}}|e(t)|dt$$ (5)

**Fig 3:**
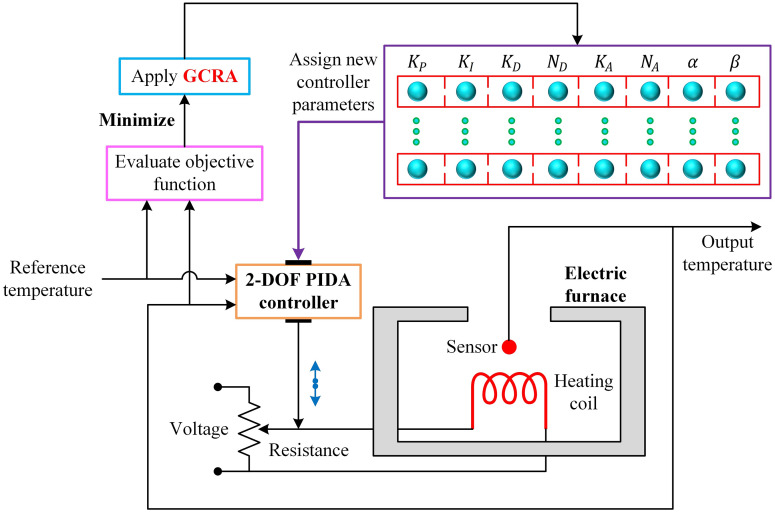
Block diagram of the GCRA-based 2DOF PIDA controlled electric furnace system.

The parameter‐tuning loop driven by the GCRA is depicted in Fig 3. At each generation, candidate controller parameters are assigned to the 2-DOF PIDA controller, and the electric‐furnace model is simulated under the prescribed reference step. The resulting output temperature is compared to the reference to form the error signal e(t), from which the objective function OF is evaluated according to Eq. (5). The GCRA then uses these OF values to guide its search (minimizing OF by balancing exploration of new parameter regions with exploitation of promising solutions) until maximum number of iterations is achieved.

## 5. Results and discussion

### 5.1. Compared algorithms and parameter settings

Five metaheuristic algorithms were evaluated to benchmark the proposed method’s performance. The greater cane rate algorithm (GCRA) [19] was included for its novel nature-inspired foraging mechanism. The polar fox optimization algorithm (PFA) [21] and hiking optimization algorithm (HOA) [22] were selected as representative of recently developed strategies demonstrating strong exploration–exploitation balance. Success-history based adaptive differential evolution with linear population size reduction (L-SHADE) [23], recognized as a winner in CEC benchmark competitions, was adopted to represent state-of-the-art differential-evolution variants. Finally, particle swarm optimization (PSO) [24], the most widely applied swarm-based technique, was used as a baseline comparison.

All algorithms were executed with their default parameter configurations to ensure an equitable comparison. A population size of 30 individuals was maintained throughout, and each optimizer was allowed 100 iterations per run. To obtain statistically meaningful results, each algorithm was independently run 25 times under these uniform settings. This standardized experimental design guaranteed that observed performance differences could be attributed to the intrinsic search behaviors of the algorithms rather than to user-tuned parameters.

### 5.2. Statistical analysis and significance testing

**Fig 4:**
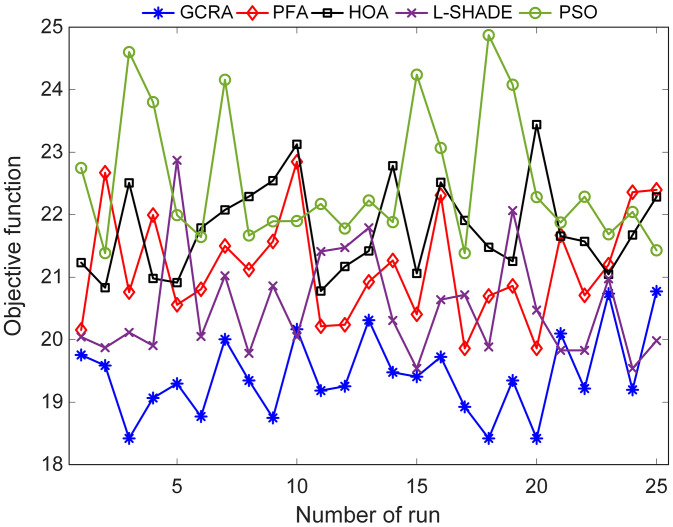
Obtained best objective function values with respect to number of runs.

To quantify and compare the optimization performance of the five algorithms, a detailed statistical evaluation was conducted. In Fig 4 the best objective‐function value obtained in each of the 25 independent runs is plotted for every method. It can be seen that the GCRA consistently achieved lower best‐of‐run values, with only rare excursions above 20. By contrast, the PSO results exhibited both higher peaks and greater dispersion, indicating less reliable convergence behavior under the same experimental conditions.

**Fig 5:**
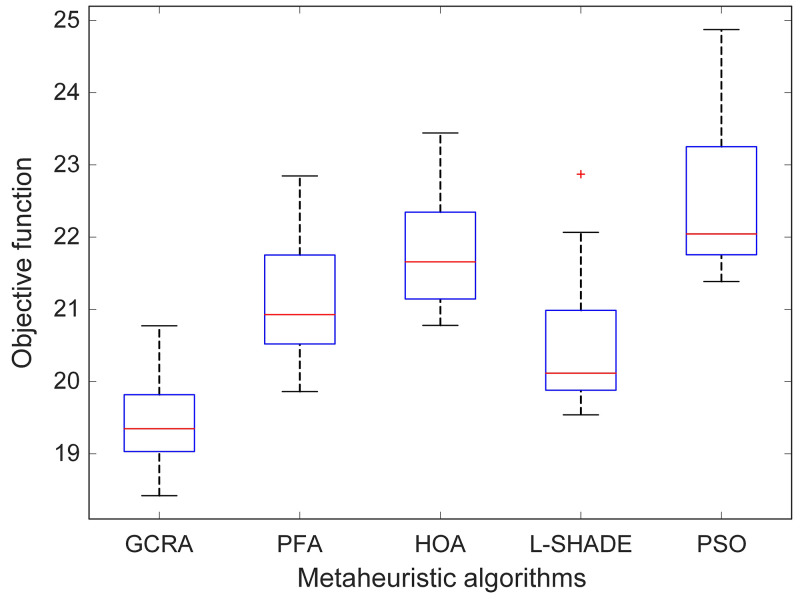
Boxplot analysis showing the distribution of objective function values with respect to number of runs.

A complementary view is provided by the box‐and‐whisker plots in Fig 5, which display the full distribution of objective‐function values across runs. The median line for GCRA lies below those of all other algorithms, and its interquartile range is the narrowest, reflecting both superior central tendency and low variability. In comparison, PFA and HOA produced higher medians and broader boxes, while L-SHADE and PSO showed still larger spreads and more pronounced outliers, suggesting that their search processes were less stable.

Numerical summaries and nonparametric significance tests are collated in Table 1. GCRA attained a minimum OF of 18.4199, a maximum of 20.7721, and an average of 19.4261 (standard deviation = 0.6582). The other methods registered higher means, e.g., PFA (21.1587), HOA (21.7733), L-SHADE (20.5208), and PSO (22.5239), with standard deviations ranging from 0.7485 to 1.0967. Pairwise Wilcoxon signed‐rank tests [33] were performed between GCRA and each comparator, yielding p-values all below 1 × 10^−^⁴. These results confirm that GCRA’s improvements over the other algorithms are statistically significant at the 5% level.

**Table 1:** Statistical performance evaluation of algorithms and p-values obtained from Wilcoxon’s test.

Statistical metric	GCRA	PFA	HOA	L-SHADE	PSO
Minimum	18.4199	19.8614	20.7778	19.5392	21.3850
Maximum	20.7721	22.8466	23.4415	22.8706	24.8729
Median	19.3464	20.9260	21.6567	20.1156	22.0437
Average	19.4261	21.1587	21.7733	20.5208	22.5239
Standard Deviation	0.6582	0.8799	0.7485	0.8578	1.0967
p-value	–	1.2290E − 05	1.2290E − 05	7.2245E − 05	1.2290E − 05

### 5.3. Objective function minimization and obtained controller parameters

**Fig 6:**
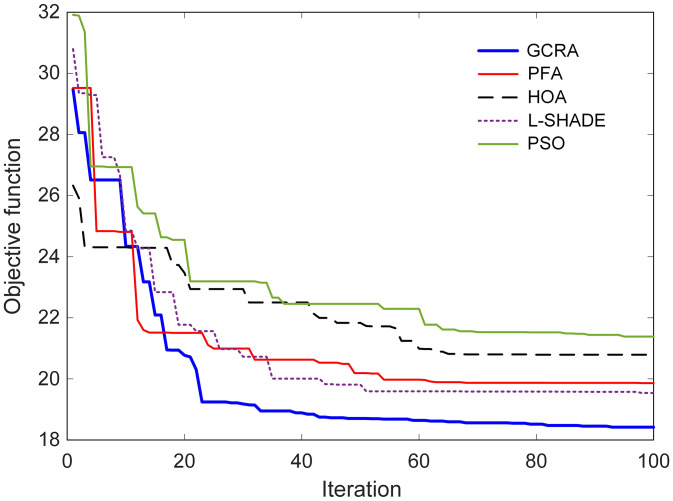
Best convergence curves of the algorithms with respect to iteration number.

The convergence behavior of each optimization method is illustrated in Fig 6, where the evolution of the objective‐function value over successive iterations is plotted for all five algorithms. It can be observed that the GCRA achieved rapid descent of the objective metric, reaching its minimum value after 93 iterations, whereas the other methods (particularly PSO and HOA) exhibited slower convergence and greater oscillation in their search trajectories.

Upon completion of the search process, the best parameter sets identified by each algorithm were recorded. Table 2 summarizes the predefined bounds for each controller parameter along with the optimal values returned by GCRA, PFA, HOA, L-SHADE, and PSO. For instance, GCRA selected a proportional gain of 2.3941 and an integral gain of 0.4060, while the derivative gain and associated filter were tuned to 3.5696 and 327.3797, respectively. The acceleration coefficient set by GCRA at 0.9993 and its associated filter was 137.8373, with feedforward weights α=1.1895 and β=1.6249. In comparison, the other algorithms returned values that deviated more markedly from the mid‐ranges of the search space, reflecting their less effective balance between global exploration and local refinement. By coupling the swift convergence displayed in Fig 6 with the parameter profiles of Table 2, it is demonstrated that GCRA not only minimizes the adaptive objective function more efficiently but also identifies controller settings that reside within well‐conditioned regions of the design space, thereby ensuring both performance and robustness in the resulting 2-DOF PIDA controller.

**Table 2:** Adopted parameter ranges and the obtained best parameters via different algorithms.

Parameter	Range	GCRA	PFA	HOA	L-SHADE	PSO
$K_{P}$	[0.1, 10]	2.3941	2.3246	2.8115	2.3148	2.5421
$K_{I}$	[0.1, 1]	0.4060	0.4506	0.5720	0.5134	0.4267
$K_{D}$	[0.1, 10]	3.5696	3.5457	3.8575	3.5234	3.1155
$N_{D}$	[5, 500]	327.3797	308.7118	164.8769	109.1211	254.6417
$K_{A}$	[0.1, 1]	0.9993	1.0969	1.0642	0.9458	0.9038
$N_{A}$	[5, 500]	137.8373	68.3960	145.0049	271.1871	133.3183
α	[0.5, 2]	1.1895	1.1835	1.0214	1.1449	1.1302
β	[0.5, 2]	1.6249	1.9786	1.2602	1.7715	1.3860

### 5.4. Transient response analysis

**Fig 7:**
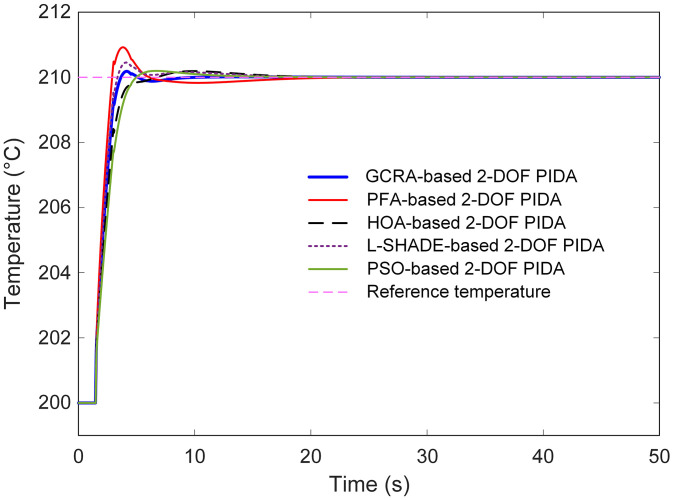
Transient response of different algorithms-based 2-DOF PIDA controllers.

**Fig 8:**
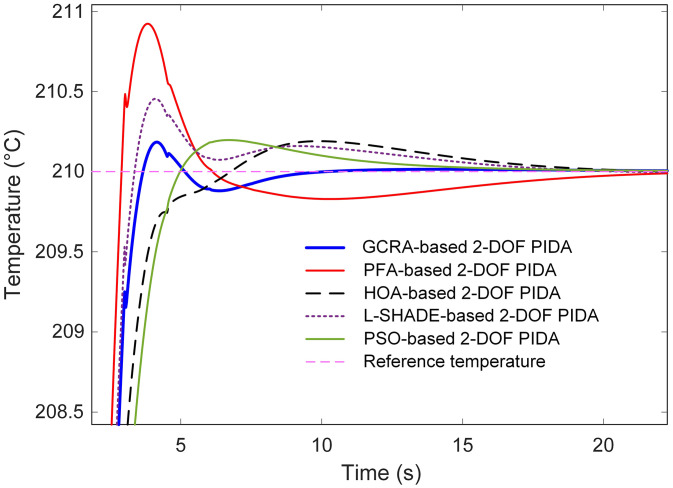
Zoomed view of Fig 7.

The dynamic behavior of the optimized 2-DOF PIDA controllers was examined through step‐response simulations. In Fig 7, the temperature trajectories following a set‐point jump from 200 °C to 210 °C are overlaid for all five algorithms. It is evident that the GCRA-tuned controller exhibits the swiftest rise toward the target, reaching the vicinity of 210 °C markedly faster than its counterparts, while PFA demonstrates the slowest approach and the largest initial overshoot. A magnified view of this interval is presented in Fig 8, where the finer distinctions in rise time and peak behavior are highlighted. The GCRA-based response settles within the ± 2 °C band in under 4 s, whereas PFA remains outside this band until approximately 6 s. HOA and PSO achieve comparable rise rates to GCRA but incur slightly larger peaks, and L-SHADE displays moderate speed yet a pronounced oscillatory tendency before settling.

**Fig 9:**
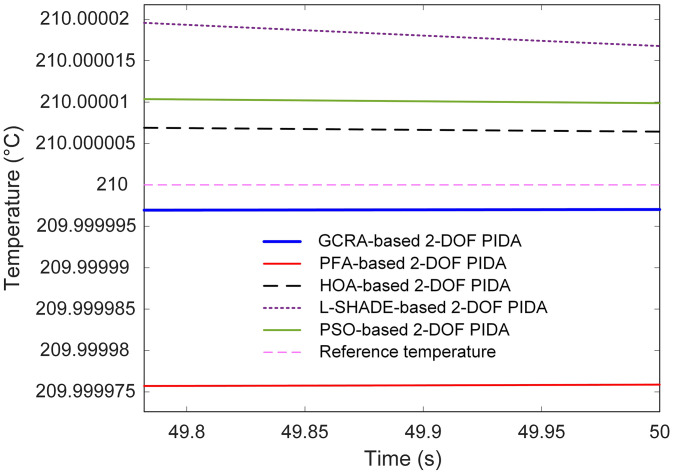
Steady-state response of different algorithms based 2-DOF PIDA controllers.

Steady-state accuracy is illustrated in Fig 9. All controllers ultimately converge close to the 210 °C set‐point; however, PFA maintains a small but visible steady‐state offset, while the GCRA and HOA variants attain virtually zero residual error. L-SHADE and PSO also approach the target precisely but require longer settling durations to do so.

These qualitative observations are substantiated by the quantitative metrics summarized in Table 3. The GCRA-optimized controller achieved the shortest settling time (3.4542 s), the lowest overshoot (1.8382%), and the smallest steady‐state error (2.9715 × 10^−5^) among all methods. In comparison, PFA required 5.3326 s to settle, exhibited a 9.2226% overshoot, and left a steady‐state error of 2.4119 × 10^−4^; HOA, L-SHADE, and PSO all registered longer settling times (4.6285 s, 5.1825 s, and 4.5496 s respectively), larger overshoots (1.8995%, 4.5433%, 1.9677%), and greater residual errors (6.4311 × 10^−5^, 1.6770 × 10^−4^, 9.8710 × 10^−5^ respectively).

**Table 3:** Time domain performance metrics of different algorithms based 2-DOF PIDA controllers.

Stability metric	GCRA	PFA	HOA	L-SHADE	PSO
Settling time (s)	**3.4542**	5.3326	4.6285	5.1825	4.5496
Peak (°C)	**210.1838**	210.9223	210.1899	210.4543	210.1968
Overshoot (%)	**1.8382**	9.2226	1.8995	4.5433	1.9677
Steady-state error (%)	**2.9715E − 05**	2.4119E − 04	6.4311E − 05	1.6770E − 04	9.8710E − 05

### 5.5. Comparisons with reported best approaches

**Fig 10:**
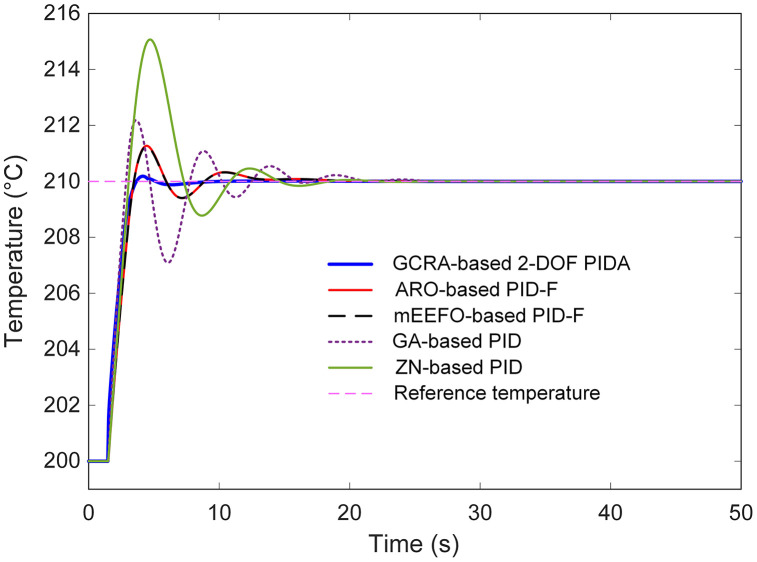
Comparative transient response analysis with respect to reported approaches….

**Fig 11:**
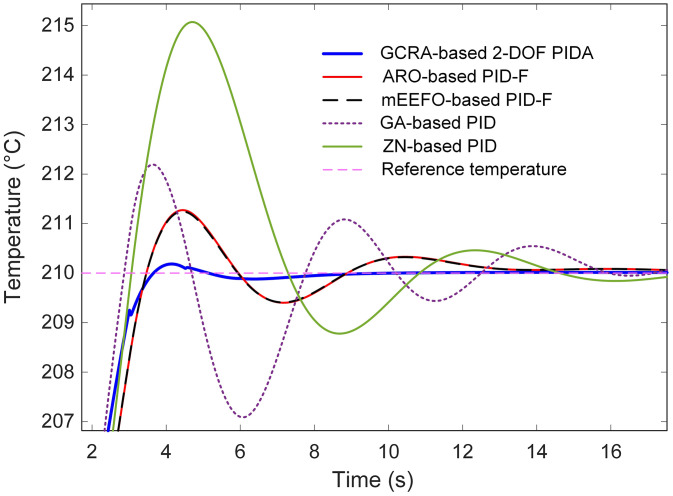
Zoomed view of Fig 10.

To contextualize the performance of the proposed GCRA-based 2-DOF PIDA controller, its results were compared against several benchmark strategies previously reported in the literature, namely the artificial rabbits optimization (ARO)-based filtered PID (PID-F) [6], modified electric eel foraging optimizer (mEEFO)-based PID-F [25], genetic algorithm (GA)-based PID [26], Ziegler-Nichols (ZN)-based PID [27] tuning method. The comparative transient responses for a step change from 200 °C to 210 °C are depicted in Fig 10, with an enlarged view in Fig 11 to better highlight early-stage dynamics. From these results, it is apparent that the GCRA-tuned 2-DOF PIDA delivers a markedly faster approach to the target temperature, achieving settling in less than 3.5 s, whereas all other reported methods required substantially longer durations. The GA- and ZN-based controllers, in particular, exhibited noticeably sluggish responses, accompanied by overshoots exceeding 20% and 50%, respectively. Both ARO- and mEEFO-based PID-F schemes showed improved transients relative to GA and ZN, yet their settling times remained above 11 s and their overshoots above 12%, underscoring their slower convergence to steady conditions.

**Fig 12:**
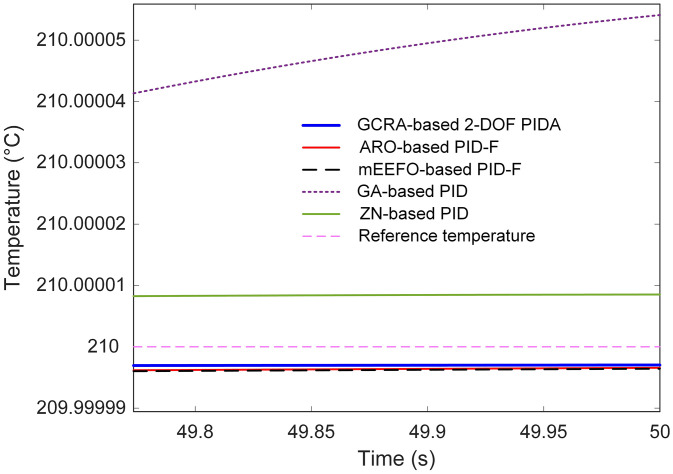
Comparative steady-state response analysis with respect to reported approaches.

Steady-state performance, presented in Fig 12, reinforces these observations. While all methods ultimately approached the set-point, the GCRA-based 2-DOF PIDA not only reached it sooner but also maintained the lowest steady-state error, recorded at 2.9715 × 10^−5^, as summarized in Table 4. In contrast, GA and ZN left higher residual errors, with the GA method exhibiting more than an order of magnitude greater steady-state deviation.

**Table 4:** Comparative time response metrics analysis with respect to reported approaches.

Stability metric	GCRA-based 2-DOF PIDA	ARO-based PID-F	mEEFO-based PID-F	GA-based PID	ZN-based PID
Settling time (s)	**3.4542**	11.8272	11.7823	19.4569	13.8103
Peak (°C)	**210.1838**	211.2718	211.2447	212.1926	215.0707
Overshoot (%)	**1.8382**	12.7180	12.4466	21.9256	50.7074
Steady-state error (%)	**2.9715E − 05**	3.4251E − 05	3.5773E − 05	5.4091E − 04	8.5227E − 05

Quantitative analysis in Table 4 further substantiates the superiority of the proposed approach. Alongside its minimal steady-state error, the GCRA-based controller attained the lowest overshoot (1.8382%) and peak temperature (210.1838 °C), whereas the ZN-based PID peaked at 215.0707 °C, far exceeding the target and inducing large oscillations before stabilization. These findings demonstrate that, by leveraging the exploration–exploitation balance of the GCRA in tuning the 2-DOF PIDA, both transient and steady-state metrics are significantly improved over those offered by the best previously reported tuning methodologies. This translates into faster, more accurate, and more stable temperature regulation, which is highly advantageous for practical thermal process control.

### 5.6. Input tracking performance analysis

**Fig 13:**
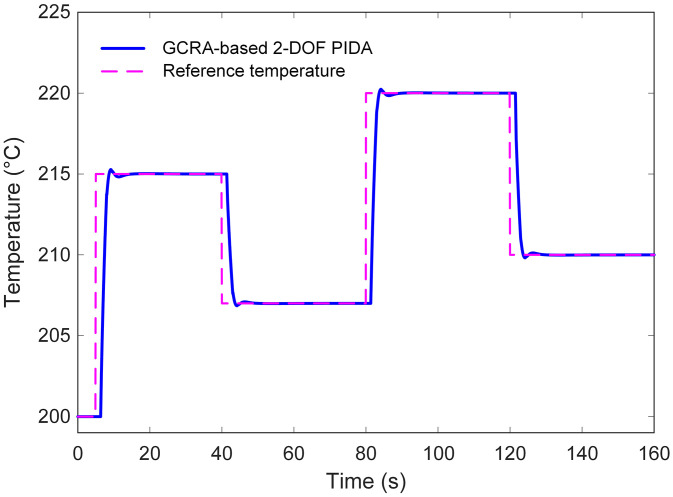
Input tracking performance analysis of the proposed approach.

The ability of the proposed GCRA‐based 2‐DOF PIDA controller to follow varying reference inputs was assessed through dedicated input‐tracking simulations. As illustrated in Fig 13, the controller was subjected to a sequence of step changes in the temperature set‐point. Across all transitions, the output closely tracked the reference trajectory, exhibiting minimal overshoot and negligible steady‐state error. This responsiveness highlights the effectiveness of the tuned control parameters in maintaining rapid adaptation without inducing instability.

**Fig 14:**
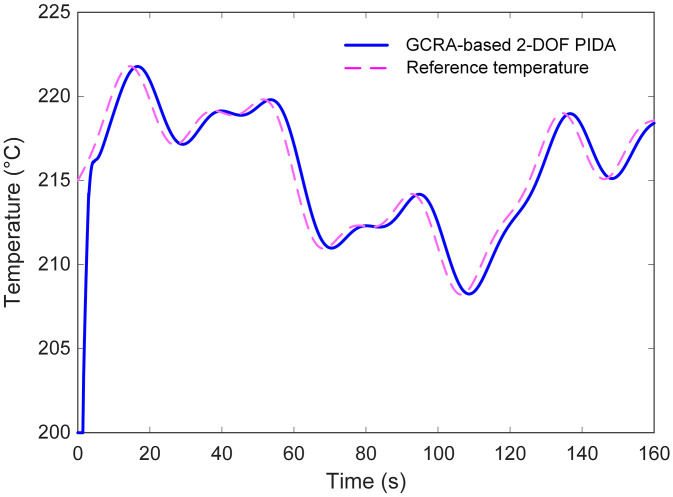
Variable input tracking performance analysis of the proposed approach.

To further examine adaptability, a variable reference profile consisting of both abrupt and gradual changes was applied, as shown in Fig 14. In this scenario, the proposed control approach maintained accurate tracking throughout, seamlessly adjusting to the different rates of change in the set‐point. The absence of oscillatory artifacts or prolonged settling periods demonstrates the controller’s robustness against dynamic variations in the desired operating point. Such consistent behavior under diverse input conditions confirms that the GCRA‐optimized 2‐DOF PIDA controller can deliver precise and reliable temperature regulation, even when the operating requirements shift over time.

### 5.7. Performance analysis for disturbance rejection and measurement noise

**Fig 15:**
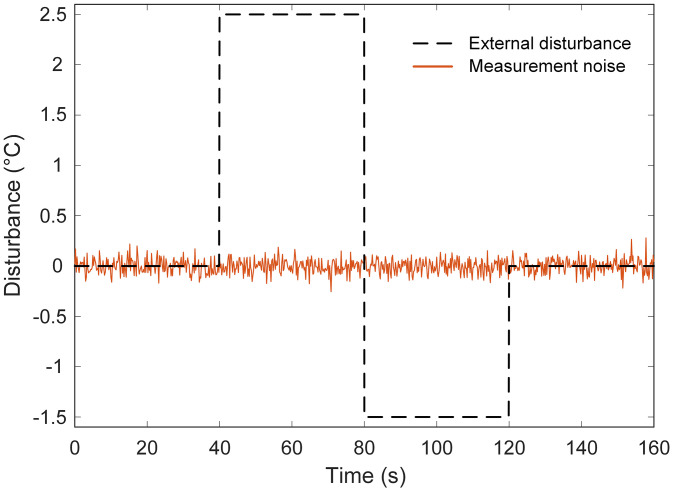
External disturbance and the measurement noise considered in this study.

The robustness of the proposed GCRA-based 2-DOF PIDA controller was further evaluated under external disturbances and measurement noise, as depicted in Fig 15. In these tests, the system was subjected to a sudden disturbance in the heater input and a superimposed Gaussian noise component on the temperature measurement signal, replicating practical operating conditions in which sensor inaccuracies and process perturbations occur simultaneously.

**Fig 16:**
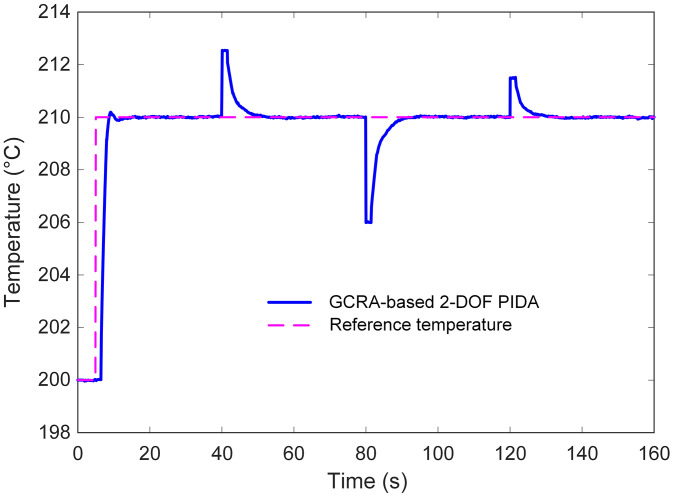
Performance of the proposed approach with respect to external disturbances and measurement noises.

The corresponding closed-loop responses are illustrated in Fig 16. It can be observed that the proposed control strategy maintained stable operation, quickly counteracting the impact of the disturbance and restoring the temperature to its nominal set-point. The corrective action was prompt, with minimal deviation from the desired value, indicating that the tuned controller parameters provided an effective balance between responsiveness and stability. Furthermore, the presence of measurement noise did not induce oscillatory artifacts or noticeable performance degradation, demonstrating strong resilience against high-frequency fluctuations. These findings confirm that the GCRA-tuned 2-DOF PIDA controller not only excels in nominal tracking tasks but also preserves performance integrity under realistic plant uncertainties. The ability to reject disturbances while suppressing noise-induced variations underscores its suitability for precise temperature regulation in electric furnace applications, where environmental and measurement-related imperfections are inevitable.

## 6. Conclusion and future work directions

In this study, a novel 2-DOF PIDA controller has been developed and applied for the first time to the temperature regulation of an electric furnace system. The proposed structure enables independent tuning of set-point tracking and disturbance rejection by introducing separate feedforward gains for proportional and derivative actions, while retaining integral and acceleration terms on the raw error signal. This structural advantage addresses the inherent limitations of conventional single-degree-of-freedom configurations, which often struggle to balance transient performance and steady-state accuracy in nonlinear, time-delayed thermal processes. The controller parameters were optimized using the GCRA, marking the first application of this recent nature-inspired metaheuristic to such a control architecture. A novel adaptive objective function, combining normalized overshoot, normalized settling time, and cumulative tracking error, was employed to achieve balanced improvements across both transient and steady-state performance measures. Comparative evaluations against state-of-the-art metaheuristic optimizers and benchmark tuning strategies demonstrated that the proposed method consistently delivered faster settling times, lower overshoot, and near-zero steady-state errors, while maintaining strong robustness under external disturbances and measurement noise. Overall, the integration of the GCRA with the 2-DOF PIDA controller offers a promising and energy-efficient solution for precise temperature regulation in industrial furnace systems, with potential applicability to other time-delay processes in different domains.

Future research directions may focus on several extensions of this work. First, experimental validation on a physical furnace setup would be valuable to confirm the real-world applicability of the proposed design under practical constraints such as actuator limitations, sensor noise, and parameter drift. Second, hybridization of the GCRA with other optimization strategies could be explored to further enhance convergence speed and global search capability. Third, adaptive or self-tuning versions of the 2-DOF PIDA controller could be developed to automatically adjust parameters in response to process variations and disturbances in real time. Additionally, extending the approach to multivariable temperature control systems and integrating predictive or learning-based elements, such as model predictive control or reinforcement learning, could further improve performance in highly dynamic and uncertain environments. These future investigations are expected to broaden the applicability and impact of the proposed methodology across a wide range of industrial control challenges.

## Abbreviations

ARO: Artificial rabbits optimization
CEC: Congress on evolutionary computation
DOF: Degree of freedom
GA: Genetic algorithm
GCRA: Greater cane rat algorithm
HOA: Hiking optimization algorithm
IAE: Integral absolute error
LB: Lower bound
PFA: olar fox algorithm
PID: Proportional–integral–derivative
PIDA: Proportional–integral–derivative–acceleration
PSO: Particle swarm optimization
SHADE: Success-history based adaptive differential evolution with linear population size reduction
UB: Upper bound
ZN: Ziegler–Nichols
